# Sex-Specific Associations between Thyroid Status, Inflammation and Hemostasis Biomarkers in Patients with Subacute Thyroiditis

**DOI:** 10.3390/biomedicines12081862

**Published:** 2024-08-15

**Authors:** Jelena Vekic, Aleksandra Klisic, Jelena Kotur-Stevuljevic, Neda Milinkovic, Sanja Gluscevic, Serpil Ciftel, Filiz Mercantepe

**Affiliations:** 1 Department for Medical Biochemistry, University of Belgrade Faculty of Pharmacy, 11000 Belgrade, Serbia; 2 Faculty of Medicine, University of Montenegro, 81000 Podgorica, Montenegro; 3 Center for Laboratory Diagnostics, Primary Health Care Center, 81000 Podgorica, Montenegro; 4 Department of Neurology, Clinical Center of Montenegro, 81000 Podgorica, Montenegro; 5 Department of Endocrinology and Metabolism, Erzurum Regional Training and Research Hospital, 25100 Erzurum, Turkey; 6 Department of Endocrinology and Metabolism, Faculty of Medicine, Recep Tayyip Erdogan University, 53200 Rize, Turkey

**Keywords:** inflammatory biomarkers, prothrombin time, activated partial thromboplastin time

## Abstract

*Background*: Subacute thyroiditis (SAT) is characterized by profound inflammation and fluctuations in thyroid hormones which may affect the hemostasis balance. This study investigates sex-specific associations between thyroid status, inflammation and hemostasis biomarkers in SAT. *Methods*: We included 52 patients (40 women and 12 men) treated with non-steroidal anti-inflammatory drugs (NSAID) or methylprednisolone (MPS). Free thyroxine (fT4), thyroid stimulating hormone, C-reactive protein, complete blood count and routine hemostasis parameters were assessed. *Results*: Both men and women were in hyperthyroid state and had comparable levels of inflammatory biomarkers. A shortened activated partial thromboplastin time (aPTT) was observed in 16.7% of the men and 10% of the women (*p* = 0.562), and a shortened prothrombin time (PT) was observed in 33% of the men and 12.5% of the women (*p* = 0.094). In men, aPTT positively correlated with fT4 (r = 0.627; *p* < 0.05), while PT positively correlated with leukocyte-based inflammatory indices in women (*p* < 0.05). NSAID-treated patients had lower aPTTs and platelet counts than those treated with MPS (*p* < 0.05). Principal component analysis extracted “proinflammatory”, “prothrombotic” and “antithrombotic” factors, but the “proinflammatory” factor was the independent predictor of elevated fT4 in women (OR = 2.705; *p* = 0.036). *Conclusions*: Our data demonstrated sex-specific associations of thyroid status and inflammatory biomarkers with hemostasis parameters in SAT. Routine hemostasis screening tests may help in monitoring the changes in the hemostasis system over the course of SAT.

## 1. Introduction

Subacute thyroiditis (SAT) is a relatively benign inflammatory disease of the thyroid gland that occurs mainly in women. To date, the exact etiology of SAT is not fully understood, but it is thought to be a complex interaction of genetic and environmental factors [1]. Although SAT is generally considered to be a self-limiting disease, in certain cases it can lead to permanent thyroid dysfunction. In recent years, interest in understanding the causes and consequences of SAT has increased, particularly following the COVID-19 pandemic [2,3,4].

In general, patients with SAT experience transient thyrotoxicosis, followed by hypothyroidism and finally complete recovery of thyroid function [1]. Available data suggest that the pleiotropic effects of thyroid hormones also extend to the hemostasis system [5]. Accordingly, fluctuations in thyroid hormone levels in SAT may disrupt the delicate balance of hemostasis by affecting the clotting and fibrinolysis processes [6], potentially increasing the risk of thrombosis or hemorrhage [7]. This may be of particular importance considering that SAT is characterized by exacerbated inflammation [8], which may contribute to the activation of the hemostasis system [9,10], as has been documented in other diseases with an inflammatory background [11,12]. However, the interplay between thyroid function, inflammation and hemostasis biomarkers in SAT is still largely unexplored.

Another important aspect of SAT is pharmacological treatment, which is primarily based on the administration of non-steroidal anti-inflammatory drugs (NSAIDs) or corticosteroids [13]. Both types of treatment can also have an effect on the hemostasis system. In particular, NSAID therapy is known to impair platelet function [14], while corticosteroid administration promotes a hypercoagulable state [15]. However, there are currently no data on the potential effects of anti-inflammatory SAT therapy on routine hemostasis biomarkers.

The aim of the present study was to evaluate the associations between thyroid function and inflammatory and routine hemostasis biomarkers in patients with SAT, stratified by gender. We also investigated the effects of SAT treatment on hemostasis screening test results and the relationship between thyroid status and hemostasis parameters at baseline with respect to SAT outcome at 6–12 months.

## 2. Materials and Methods

### 2.1. Study Participants

This study comprised 52 patients, including 40 women and 12 men. The coagulation parameters of these patients were examined as an extension of a previously published study on people diagnosed with SAT [8]. The patients were monitored and treated at the Endocrinology and Metabolic Diseases Outpatient Clinic of Erzurum Teaching and Research Hospital between November 2021 and June 2022. The diagnosis of SAT was made in all patients based on the presence of characteristic symptoms, physical examination findings, increased erythrocyte sedimentation rate (ESR), increased free thyroxine (fT4) levels, decreased thyroid stimulating hormone (TSH) levels, decreased radioactive iodine uptake, and the identification of hypoechoic regions with blurred borders and decreased vascularization in thyroid ultrasound. Patients were monitored for 6–12 months. All patients were treated with either indomethacin or methylprednisolone (MPS) at a dosage of 16–32 mg, depending on their clinical condition. The method of adjusting the dosage and duration of treatment may vary depending on the patient’s specific clinical symptoms. All subjects included in the study suffered from SAT only and did not undergo any other medical intervention besides treatment specifically for SAT. In addition, patients who were under 18 years of age, pregnant, breastfeeding, diagnosed with postpartum thyroiditis or suffering from amiodarone thyroiditis were excluded from the study. The study was conducted in accordance with the ethical principles of the Declaration of Helsinki and written informed consent was obtained from all participants.

### 2.2. Laboratory Methods

The biochemical and hematological examinations were performed in the morning between 08:00 and 09:00 after a fasting period of at least eight hours. Complete blood count parameters, including white blood cell count, platelet count, neutrophil count, lymphocyte count and monocyte count, were analyzed using a Sysmex XN 9000 Autoanalyzer (Kobe, Japan). Prothrombin time (PT) and activated partial thromboplastin time (aPTT) were determined using a Sysmex CA-660 automatic hemostasis analyzer (Kobe, Japan). A Cobas C 701 biochemical autoanalyzer (Roche, Mannheim, Germany) was used to examine other biochemical parameters. A chemiluminescent immunoassay (Beckman Coulter, DXI 800, Brea, CA, USA) was used to quantify thyroid stimulating hormone (TSH) and free thyroxine (fT4). The reference ranges for fT4, TSH, ESR and CRP were set as follows: fT4: 11.46–22.65 pmol/L, TSH: 0.55–4.78 mIU/L, ESR: 0–20 mm/hour and CRP: 0–5 mg/L. The leukocyte-based indices were calculated as follows: NLR by dividing the number of neutrophils by the number of lymphocytes; PLR by dividing the number of platelets by the number of lymphocytes; SII by multiplying the number of neutrophils by the number of platelets and then dividing by the number of lymphocytes; SLR by dividing the ESR by the number of lymphocytes; LMR by dividing the number of lymphocytes by the number of monocytes; TLR by dividing the number of platelets by the number of lymphocytes; CLR by dividing the number of platelets by the number of lymphocytes; and GLR by dividing the glucose level by the number of lymphocytes.

### 2.3. Statistical Analysis

The distribution of the data was tested using the Shapiro–Wilk test. Results are presented as mean and 95% confidence interval (CI) for means for data not significantly deviating from a normal distribution and as median and interquartile range (25th to 75th percentile) for data not normally distributed. Differences between groups were analyzed using the Mann–Whitney U test. Categorical variables were compared using the chi-square test, while correlations were analyzed using Spearman’s rank correlation coefficients. Principal component analysis (PCA) with varimax-normalized rotation was performed to extract the factors, that were then tested using binary logistic regression analysis. Specifically, an eigenvalue >1 was used to extract the factors, while variables with factor loadings >0.5 were considered for the interpretation of the selected factors in PCA. Binary logistic regression analysis was used to test for a possible independent association between selected factors and highly increased fT4 values, i.e., values above the 75th percentile (fT4 > 30.5 pmol/L). Statistical analysis was performed using PASW^®^ Statistic v.18 software (Chicago, IL, USA), and *p*-values < 0.05 were considered to indicate statistical significance.

## 3. Results

The current study included 52 consecutive patients with SAT aged 43 (40–47) years. Although gender was not an exclusion criterion, the majority of patients were female (76.9%). The men were on average 48 (39–58) years old, and the women, 42 (39–46) years old (*p* = 0.385). The average SAT recovery time was 30 (20–40) days, with no significant difference between the sexes (*p* = 0.501). A statistically significant difference (*p* < 0.05) was found between the number of men and women treated with NSAIDs (34.4% men vs. 66.6% women) and MPS (5% men vs. 95% women).

The results of the laboratory analyses are shown in Table 1. There were no significant differences in the number of neutrophils and lymphocytes between men and women. However, the monocyte count was significantly higher in men. There were no differences between the sexes in routine hemostasis parameters (PT, aPTT and INR) or platelet count. A shortened aPTT (<25 s) was observed in 16.7% of men and 10% of women (*p* = 0.562), while PT was shortened in 33% of men and 12.5% of women (<12 s) (*p* = 0.094). The analysis of biochemical parameters revealed a slight reduction in HDL-C and TG levels in SAT patients, and these changes were more pronounced in men. As expected, the parameters of thyroid status indicated hyperthyroidism, with no gender differences. Similarly, men and women with SAT had comparable levels of inflammatory biomarkers (Table 2). However, the difference in the monocyte/HDL-C ratio was of borderline significance (*p* = 0.050), as the monocyte count was significantly higher in men.

In all SAT patients, higher CRP levels were associated with increased fT4 and reduced TSH levels. The fT4 levels were also positively related to lymphocyte-based inflammatory indices, while TSH levels showed opposite relationships, with the exception of LMR. In addition, THS levels showed no correlations with SII and NLR indices (Table 3). Of note, no significant correlations were found with hemostasis parameters.

We then examined the relationships between inflammatory parameters, thyroid status and hemostasis in relation to gender (Table 4). In men, TSH levels were inversely associated with INR, while fT4 levels were positively correlated with aPTT. Of the inflammatory biomarkers examined, only NLR was inversely associated with aPTT. Platelet count in men was positively correlated with ESR and SII. In contrast, no significant correlations were found between thyroid status and hemostasis parameters in women. However, PT and INR were positively correlated with the inflammatory indices SII and NLR in women, while PT was also positively correlated with neutrophil count. In addition, positive correlations were found between platelet count and neutrophil and monocyte counts, as well as with the SII and NLR indices. The platelet count in women was also positively associated with the CRP level. As expected, the associations between fT4 levels and inflammatory biomarkers observed in the study group as a whole were also seen in the separate analysis of women.

Regarding associations with biochemical parameters, we found that the HDL-C level was inversely associated with INR in men (r = −0.820; *p* < 0.05), while serum glucose level was positively correlated with fT4 level in women (r = 0.328; *p* < 0.05).

**Figure 1:**
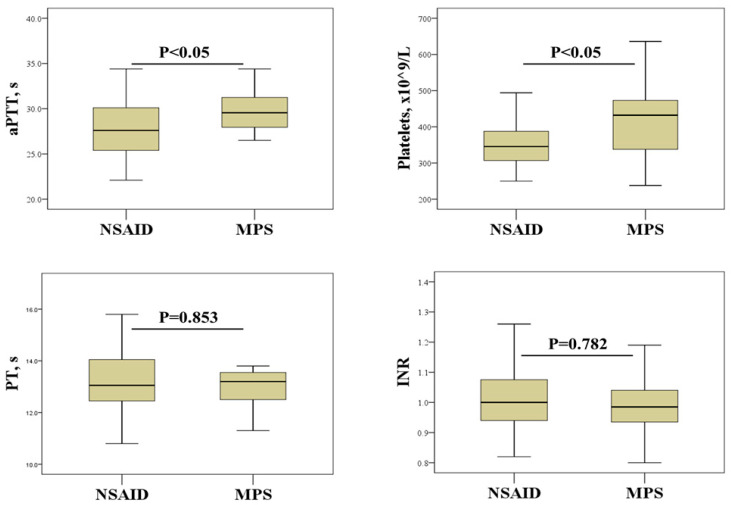
Effect of SAT treatment on hemostasis parameters and platelet count. NSAIDs, non-steroidal anti-inflammatory drugs; MPS, methylprednisolone; aPTT, activated partial thromboplastin time; PT, prothrombin time; INR, international normalized ratio.

We further investigated the effects of SAT treatment on hemostasis screening and platelet count (Figure 1). This analysis revealed that SAT patients treated with NSAIDs had significantly lower aPTTs and platelet counts than patients treated with MPS.

In order to test for a possible independent association between the investigated laboratory variables and highly increased fT4 levels, we first reduced the number of parameters by PCA. The factors extracted by PCA consisted of parameters with comparable variability. The Kaiser–Meyer–Olkin index (KMO) as a measure of sampling adequacy was 0.521 (the condition for KMO > 0.500 is satisfied), and the Bartlett’s test for sphericity, which yielded *p* = 0.001 (the condition for Bartlett’s test *p* is less than 0.05), demonstrated the significance of the analysis. The first principal component is the linear combination of variables that accounts for the largest proportion of variance in the data, the second principal component is the combination that accounts for the next largest proportion, and so on. The extracted factors with the loadings of the included variables are shown in Table 5.

The extracted factors explained 46% of the total variance of the included parameters. The first factor was labelled “proinflammatory factor” and included the following: anti-inflammatory therapy; CRP level, platelet, neutrophil and monocyte counts; and ESR, all with positive loadings. This factor explained 19% of the total variance. The second factor explained 15% of the total variance and was termed the “antithrombotic factor” due to its negative loadings for aPTT and HDL-C and its positive loading for glucose. The third factor explained 12% of the variance and was interpreted as a “prothrombotic factor” as it loaded positively for gender and TG and negatively for PT.

Scores for each factor were calculated and used as independent variables in a binary logistic regression analysis to determine associations with highly increased fT4 levels (fT4 > 30.5 pmol/L). As shown in Table 6, among the extracted factors, the only significant predictor of fT4 levels was “proinflammatory factor” in a group of female patients. Specifically, this analysis showed that increased fT4 levels were predicted by a higher value of “proinflammatory factor” (OR = 2.705; *p* = 0.036). This regularity did not apply to the group of male patients.

Although 13.5% of patients developed permanent thyroid dysfunction during the follow-up period, we found no significant differences in inflammatory or hemostasis parameters at baseline between patients who developed hypothyroidism and those who did not. However, in patients who developed permanent hypothyroidism during follow-up, baseline PT was positively associated with fT4 level (r = 0.893, *p* < 0.01), but inversely associated with TSH level (r = −0.867, *p* < 0.01). It is noteworthy that such correlations were not observed in euthyroid patients.

## 4. Discussion

In the present study, we were able to demonstrate gender-specific associations between thyroid status, inflammatory biomarkers and routine parameters of hemostasis in patients with SAT. In particular, significant associations between inflammatory and hemostasis biomarkers were found in women, while an association between thyroid status and hemostasis biomarkers was evident in men. Furthermore, the “proinflammatory factor” was found to be a significant predictor of elevated fT4 levels only in women. Furthermore, our data showed differential effects of anti-inflammatory treatment of SAT on the results of routine hemostasis tests.

Highly elevated inflammatory biomarkers are one of the main features of SAT, with ESR and CRP being measured most frequently. These characteristic findings were also confirmed in the current study, along with an increase in several novel leukocyte-based inflammatory indices (Table 2), as observed by others [8,16,17]. Although there were no gender differences in the inflammatory markers studied, the monocyte count was significantly higher in men (Table 1). Sexual dimorphism in leukocyte composition has previously been reported, with men having higher monocyte counts [18], although the exact role of testosterone is not fully understood [19,20]. The patients included in our study were hyperthyroid, as evidenced by increased fT4 levels and decreased TSH (Table 1). Furthermore, elevated fT4 levels correlated with increased inflammatory biomarkers (Table 3), reflecting the inflammatory response and damage to thyroid tissue.

Although hemostasis screening is not routinely performed in patients with SAT, the systemic effects of inflammation and fluctuations in thyroid hormone levels during the course of SAT can disrupt the balance between plasma procoagulants and anticoagulants. An interesting finding observed in men was a positive correlation between fT4 levels and aPTT (Table 4), suggesting a tendency for prolonged clotting time with elevated thyroid hormones. Of note, aPTT is responsive to a lack of contact factors and intrinsic and common pathway factors, but can also be prolonged in inflammatory patients [21]. However, in the vast majority of our SAT patients, aPTT was within the reference values, indicating that further investigation is needed to clarify the mechanisms behind the observed association. Nevertheless, a certain number of patients had a shortened PT and aPTT, implying that hemostasis screening may identify those with a prothrombotic state during the hyperthyroid phase. On the other hand, hypothyroidism occurring in the latter phase of SAT may lead to a bleeding tendency [22]. Taken together, our data suggest that routine hemostasis screening tests may help to monitor changes in the hemostasis system between the hyperthyroid and hypothyroid phases of SAT.

In addition to producing an excess of thyroid hormones, inflammation can also alter the concentration of coagulation factors [9], which can influence the results of hemostasis screening tests. In the current study, a positive association was found between PT and the inflammatory biomarkers SII and NLR, but only in women (Table 4). At this point, it is important to mention that our data showed different effects of anti-inflammatory treatment of SAT on hemostasis biomarkers (Figure 1). Although NSAID therapy has no direct effects on hemostasis screening tests, its effects on platelet function could indirectly influence the results of PT and aPTT tests [23]. The lack of a significant association between thyroid parameters and hemostasis parameters in women (Table 4) suggests that inflammation rather than thyroid dysfunction contributes to the disturbances in hemostasis in women with SAT. 

Thyroid disorders are associated with a number of abnormalities in platelet count, morphology and function [7,24,25,26]. The results of the current study suggest that platelet count may be a more reliable indicator of the procoagulant state associated with inflammation in SAT. Indeed, platelet count showed a significant positive correlation with most of the inflammatory biomarkers examined, including ESR, CRP, and neutrophil and lymphocyte counts (Table 4). This is not an unexpected result considering that platelets, in addition to their hemostatic function, also play an important role in regulating immune response and inflammation [27]. Another important finding of our study is the effects of SAT therapy on platelets. In particular, a significantly higher platelet count was found in patients treated with corticosteroids than in patients receiving NSAIDs (Figure 1). Although previous studies have shown the effects of corticosteroid and NSAID therapy on platelet count and function [14,15], to our knowledge, this study is the first to demonstrate such effects in patients with SAT. Elevated platelet counts in patients treated with corticosteroids indicate a potential risk of developing a hypercoagulable state. This may be of particular importance in women as they tend to have higher platelet reactivity than men [28,29]. However, some studies suggest that corticosteroid therapy can prevent the development of permanent hypothyroidism after SAT, emphasizing the need to weigh the risks and benefits [30,31].

The associations reported in the current study indicate multiple interactions between biomarkers of pathophysiological processes in SAT, with the interplay between inflammation and thyroid dysfunction being the most prominent (Table 3). The mutual associations and common features of the studied biomarkers were further revealed by PCA, which categorized them into different phenotypes such as “proinflammatory”, “prothrombotic” and “antithrombotic” phenotypes (Table 5). Among these, the “proinflammatory factor” was found to be an independent predictor of elevated fT4 in women, confirming the central role of inflammation in the development of hyperthyroidism in SAT (Table 6).

Our study has several limitations. First, the design of our study did not allow us to examine a causal relationship between SAT and hemostasis disorders in later life. Longitudinal studies tracking changes in biomarkers of thyroid function, inflammation and hemostasis during the course of SAT and after clinical remission are needed to determine the dynamics of these associations and their long-term effects. Furthermore, since all patients received anti-inflammatory therapy, our results and conclusions cannot be extrapolated to untreated patients. Another limitation is the lack of data on specific coagulation factors and platelet indices, such as mean platelet volume, platelet volume distribution and platelet crit. The inclusion of these parameters would provide further insight into the integrity of the coagulation process and platelet functionality in SAT patients. Nevertheless, the results of our current study may serve as a basis for future research. Finally, this study was performed in a relatively small number of patients, so future studies with a larger sample size are needed to investigate the significance of the observed results.

## 5. Conclusions

The data presented here indicate gender-specific associations between thyroid status, inflammation and hemostasis biomarkers in patients with SAT. Our results suggest that comprehensive laboratory testing, including hemostasis biomarkers, may be useful in identifying SAT patients who should be monitored more closely for potential complications. In this way, the routine screening of hemostasis and platelet count may be suggested in the case of corticosteroid treatment and permanent thyroid dysfunction. Further studies to investigate the observed complex interplay between gender, inflammation, thyroid function and hemostasis are needed to improve the treatment of SAT in both men and women.

## Figures and Tables

**Table 1:** Biochemical and hematological parameters in patients with SAT.

Parameter	All (N = 52)	Men (N = 12)	Women (N = 40)	P_gender_
Neutrophils, ×10^9^/L *	5.9 (5.1–6.6)	6.2 (4.0–8.4)	5.8 (5.0–6.7)	0.487
Lymphocytes, ×10^9^/L *	2.0 (1.8–2.2)	2.3 (1.8–2.7)	1.9 (1.8–2.1)	0.441
Monocytes, ×10^9^/L *	0.66 (0.57–0.83)	0.86 (0.48–1.24)	0.67 (0.60–0.74)	0.025
Platelets, ×10^9^/L *	354 (312–472)	386 (263–509)	394 (354–434)	0.753
PT, s	13.2 (12.5–13.8)	13.9 (12.1–15.8)	13.2 (12.5–13.7)	0.794
INR	1.0 (0.9–1.1)	1.0 (0.9–1.1)	1.0 (0.9–1.1)	0.328
aPTT, s *	28.7 (27.6–29.7)	28.7 (24.7–32.9)	28.6 (27.6–29.7)	0.558
Glucose, mmol/L	5.4 (4.7–6.1)	4.2 (4.1–4.8)	5.6 (4.7–6.1)	0.891
HDL-C, mmol/L	1.16 (0.91–1.24)	0.91 (0.85–0.93)	1.16 (0.96–1.34)	0.139
TG, mmol/L *	1.33 (0.88–1.65)	0.98 (0.55–1.4)	1.36 (1.04–1.83)	0.091
TSH, mIU/L	0.008 (0.008–0.010)	0.008 (0.008–0.008)	0.008 (0.008–0.115)	0.435
fT4, pmol/L *	27.0 (19.3–30.9)	33.5 (16.7–50.2)	25.7 (18.0–29.6)	0.178

The data are presented as medians (interquartile ranges) and were compared using the Mann–Whitney U test. * Data are presented as the mean (95% CI). PT, prothrombin time; INR, international normalized ratio; aPTT, activated partial thromboplastin time; HDL-C, high-density lipoprotein cholesterol; TG, triglyceride; TSH, thyroid stimulating hormone; fT4, free thyroxine.

**Table 2:** Inflammatory biomarkers in patients with SAT.

Parameter	All (N = 52)	Men (N = 12)	Women (N = 40)	P_gender_
CRP, mg/L *	60 (32–91)	70.5 (26.9–114)	60 (32.5–85)	0.712
ESR, mm/h *	45 (30–67)	45 (25–65)	49 (31–71)	0.450
SII *	1190 (1016–1363)	1033 (695–1372)	1223 (1120–1425)	0.529
NLR	3.0 (2.6–3.4)	2.2 (2.1–2.4)	2.9 (2.4–3.4)	0.617
TLR *	205 (189–228)	175 (103–247)	211 (187–236)	0.257
LMR	2.76 (2.26–3.92)	2.91 (1.90–3.93)	2.76 (2.25–3.59)	0.241
GLR *	2.9 (2.6–3.2)	2.4 (1.6–3.2)	3.0 (2.7–3.3)	0.298
CLR *	28 (17–44)	30.5 (11.4–49.6)	28 (17.5–43.5)	0.983
Monocytes/HDL-C *	0.016 (0.012–0.020)	0.023 (0.010–0.037)	0.015 (0.012–0.019)	0.050
ESR/Lymphocytes	22 (16–33)	13.5 (12–16.5)	25 (17–33)	0.190

The data are presented as medians (interquartile ranges) and were compared using the Mann–Whitney U test. * Data are presented as the mean (95% CI). CRP, C-reactive protein; ESR, erythrocyte sedimentation rate; SII, systemic immune-inflammation index; NLR, neutrophil-to-lymphocyte ratio; TLR, thrombocyte-to-lymphocyte ratio; LMR, lymphocyte-to-monocyte ratio; GLR, glucose-to-lymphocyte ratio; CLR, CRP-to-lymphocyte ratio.

**Table 3:** Associations between inflammation and thyroid status parameters in patients with SAT.

	fT4, pmol/L		TSH, mUI/L	
Parameter	r	*p*	r	*p*
CRP, mg/L	0.380	<0.01	−0.372	<0.01
SII	0.333	<0.05	−0.128	0.367
NLR	0.313	<0.05	−0.086	0.546
LMR	−0.351	<0.05	0.284	<0.05
CLR	0.417	<0.01	−0.372	<0.01
Monocytes/HDL-C	0.317	<0.05	−0.411	<0.01

The results of Spearman’s correlation analysis. TSH, thyroid stimulating hormone; CRP, C-reactive protein; LMR, lymphocyte-to-monocyte ratio; HDL-C, high-density lipoprotein cholesterol, NLR, neutrophil-to-lymphocyte ratio; SII, systemic immune-inflammation index, CLR, CRP-to-lymphocyte ratio.

**Table 4:** Associations between inflammation, thyroid status and hemostasis parameters in relation to gender.

Parameter	Sex	PT, s	aPTT, s	INR	Platelets, ×10^9^/L
TSH, mUI/L	Men	−0.452	−0.524	−0.604 *	0.059
	Women	0.036	0.117	0.084	−0.210
fT4,pmol/L	Men	−0.119	0.627 *	0.238	0.028
	Women	−0.109	−0.018	−0.209	0.269
ESR, mm/h	Men	−0.446	−0.114	−0.455	0.745 **
	Women	−0.171	−0.184	−0.214	0.182
Neutrophils, ×10^9^/L	Men	−0.070	−0.140	0.112	0.315
	Women	0,326 *	0.086	0.288	0.438 **
Lymphocytes, ×10^9^/L	Men	0.277	0.070	0.266	0.119
	Women	0.005	−0.108	−0.089	0.084
Monocytes, ×10^9^/L	Men	0.011	−0.135	0.277	0.337
	Women	0.256	−0.064	0.259	0.514 **
CRP, mg/L	Men	0.249	0.231	0.559	0.336
	Women	−0.294	0.055	−0.243	0.317 *
SII	Men	−0.175	−0.392	0.056	0.741 **
	Women	0.382 *	0.063	0.397 *	0.696 **
NLR	Men	−0.112	−0.600 *	−0.102	−0.032
	Women	0.347 *	0.151	0.393 *	0.347 *

The results of Spearman’s correlation analysis: * *p* < 0.05; ** *p* < 0.01. TSH, thyroid stimulating hormone; ESR, erythrocyte sedimentation rate; CRP, C-reactive protein; SII, systemic immune-inflammation index; NLR, neutrophil-to-lymphocyte ratio.

**Table 5:** PCA-derived factors in SAT patients.

Factors	Variables Included in the Factor	Loadings of the Variables	Factor Variability, % (Total Variance: 46%)
Proinflammatory factor	Anti-inflammatory therapy	0.702	19
	CRP, mg/L	0.687	
	Platelets, ×10^9^/L	0.645	
	Neutrophils, ×10^9^/L	0.622	
	Monocytes, ×10^9^/L	0.619	
	ESR, mm/h	0.574	
Antithrombotic factor	aPTT, s	−0.669	15
	HDL-C, mmol/L	−0.578	
	Glucose, mmol/L	0.546	
Prothrombotic factor	Gender	0.635	12
	PT, s	−0.536	
	TG, mmol/L	0.522	

CRP, C-reactive protein; ESR, erythrocyte sedimentation rate; APTT, activated partial thromboplastin time; HDL-C, high-density lipoprotein cholesterol; PT, prothrombin time; TG, triglycerides.

**Table 6:** Logistic regression analysis for the prediction of increased fT4 levels by PCA-derived factors in women.

Predictors	B (SE)	Wald Coefficient	OR (95% CI)	*p*
Proinflammatory factor	0.995 (0.476)	4.375	2.705 (1.065–6.875)	0.036
Antithrombotic factor	−0.285 (0.506)	0.319	0.752 (0.279–2.025)	0.573
Prothrombotic factor	−0.751 (0.673)	1.244	0.472 (0.126–1.766)	0.265

SE, standard error; OR, odds ratio; CI, confidence interval.

## Data Availability

The data will be available upon reasonable request (contact person: aleksandranklisic@gmail.com).

## References

[1] Stasiak M., Lewiński A. (2021). New Aspects in the Pathogenesis and Management of Subacute Thyroiditis. Rev. Endocr. Metab. Disord..

[2] Ciftel S., Tüzün Z. (2023). Subacute Thyroiditis Following SARS-CoV-2 Vaccination: An Autoimmune/Inflammatory Syndrome Induced by Adjuvants (Asia Syndrome). Acta Endocrinol..

[3] Fallahi P., Elia G., Ragusa F., Paparo S.R., Patrizio A., Balestri E., Mazzi V., Benvenga S., Varricchi G., Gragnani L. (2023). Thyroid Autoimmunity and SARS-CoV-2 Infection. J. Clin. Med..

[4] Szklarz M., Gontarz-Nowak K., Kieroński A., Golon K., Górny J., Matuszewski W., Bandurska-Stankiewicz E. (2024). The Co-Occurrence of SAT, Hypophysitis, and Schnitzler Syndrome after COVID-19 Vaccination: The First Described Case. Hormones.

[5] Davis P.J., Mousa S.A., Schechter G.P. (2018). New Interfaces of Thyroid Hormone Actions with Blood Coagulation and Thrombosis. Clin. Appl. Thromb..

[6] Akinci B., Comlekci A., Ozcan M.A. (2011). The Alteration of Coagulation in Patients with Thyroid Dysfunction. Recent Pat. Endocr. Metab. Immune Drug Discov..

[7] Elbers L.P.B., Fliers E., Cannegieter S.C. (2018). The Influence of Thyroid Function on the Coagulation System and Its Clinical Consequences. J. Thromb. Haemost..

[8] Çiftel S., Tüzün Z. (2023). Could the Systemic Immune Inflammation Index Predict Diagnosis, Recovery Time, Hypothyroidism, and Recurrence Rates in Subacute Thyroiditis?. Int. J. Gen. Med..

[9] Foley J.H., Conway E.M. (2016). Cross Talk Pathways between Coagulation and Inflammation. Circ. Res..

[10] Petäjä J. (2011). Inflammation and Coagulation. An Overview. Thromb. Res..

[11] Stark K., Massberg S. (2021). Interplay between Inflammation and Thrombosis in Cardiovascular Pathology. Nat. Rev. Cardiol..

[12] Rafaqat S., Gluscevic S., Patoulias D., Sharif S., Klisic A. (2024). The Association between Coagulation and Atrial Fibrillation. Biomedicines..

[13] Ray I., D’Souza B., Sarker P., Agarwal P. (2022). Management of Subacute Thyroiditis—A Systematic Review of Current Treatment Protocols. Int. J. Gen. Med..

[14] Tsoupras A., Gkika D.A., Siadimas I., Christodoulopoulos I., Efthymiopoulos P., Kyzas G.Z. (2024). The Multifaceted Effects of Non-Steroidal and Non-Opioid Anti-Inflammatory and Analgesic Drugs on Platelets: Current Knowledge, Limitations, and Future Perspectives. Pharmaceuticals.

[15] Pofi R., Caratti G., Ray D.W., Tomlinson J.W. (2023). Treating the Side Effects of Exogenous Glucocorticoids; Can We Separate the Good from the Bad?. Endocr. Rev..

[16] Calapkulu M., Sencar M.E., Sakiz D., Duger H., Ozturk Unsal I., Ozbek M., Cakal E. (2020). The Prognostic and Diagnostic Use of Hematological Parameters in Subacute Thyroiditis Patients. Endocrine.

[17] He P., Yang H., Lai Q., Kuang Y., Huang Z., Liang X., Huang H., Qin Y., Luo Z. (2022). The Diagnostic Value of Blood Cell-Derived Indexes in Subacute Thyroiditis Patients with Thyrotoxicosis: A Retrospective Study. Ann. Transl. Med..

[18] Chen Y., Zhang Y., Zhao G., Chen C., Yang P., Ye S., Tan X. (2016). Difference in Leukocyte Composition between Women before and after Menopausal Age, and Distinct Sexual Dimorphism. PLoS ONE.

[19] Brand J.S., Van der Schouw Y.T., Dowsett M., Folkerd E., Luben R.N., Wareham N.J., Khaw K.T. (2012). Testosterone, SHBG and Differential White Blood Cell Count in Middle-Aged and Older Men. Maturitas.

[20] Gagliano-Jucá T., Pencina K.M., Guo W., Li Z., Huang G., Basaria S., Bhasin S. (2020). Differential Effects of Testosterone on Circulating Neutrophils, Monocytes, and Platelets in Men: Findings from Two Trials. Andrology.

[21] Van Rossum A.P., Vlasveld L.T., van den Hoven L.J.M., de Wit C.W.M., Castel A. (2012). False Prolongation of the Activated Partial Thromboplastin Time (APTT) in Inflammatory Patients: Interference of C-Reactive Protein. Br. J. Haematol..

[22] Gao F. (2016). Variation Tendency of Coagulation Parameters in Different Hypothyroidism Stages. Acta Endocrinol..

[23] Driver B., Marks D.C., van der Wal D.E. (2020). Not All (N)SAID and Done: Effects of Nonsteroidal Anti-Inflammatory Drugs and Paracetamol Intake on Platelets. Res. Pract. Thromb. Haemost..

[24] Colonnello E., Criniti A., Lorusso E., Curreli M., Santulli M., Angeloni A., Gnessi L., Gandini O., Lubrano C. (2023). Thyroid Hormones and Platelet Activation in COVID-19 Patients. J. Endocrinol. Invest..

[25] Cao Y.T., Zhang K.Y., Sun J., Lou Y., Lv T.S., Yang X., Zhang W.H., Yu J.Y., Wu Q.B., Zhou X.Q. (2022). Platelet Abnormalities in Autoimmune Thyroid Diseases: A Systematic Review and Meta-Analysis. Front. Immunol..

[26] Ordookhani A., Burman K.D. (2017). Hemostasis in Hypothyroidism and Autoimmune Thyroid Disorders. Int. J. Endocrinol. Metab..

[27] Chen Y., Zhong H., Zhao Y., Luo X., Gao W. (2020). Role of Platelet Biomarkers in Inflammatory Response. Biomark. Res..

[28] Ranucci M., Aloisio T., Di Dedda U., Menicanti L., de Vincentiis C., Baryshnikova E. (2019). Gender-Based Differences in Platelet Function and Platelet Reactivity to P2Y_12_ Inhibitors. PLoS ONE.

[29] de Gaetano G., Bonaccio M., Cerletti C. (2023). How Different Are Blood Platelets from Women or Men, and Young or Elderly People?. Haematologica.

[30] Sencar M.E., Calapkulu M., Sakiz D., Hepsen S., Kus A., Akhanli P., Unsal I.O., Kizilgul M., Ucan B., Ozbek M. (2019). An Evaluation of the Results of the Steroid and Non-Steroidal Anti-Inflammatory Drug Treatments in Subacute Thyroiditis in Relation to Persistent Hypothyroidism and Recurrence. Sci. Rep..

[31] Gokkaya N., Tezcan K.A. (2024). The Effects of Corticosteroid and Nonsteroid Anti-Inflammatory Therapies on Permanent Hypothyroidism Occurring After the Subacute Thyroiditis. Endocr. Res..

